# Pin orthosis extension block pinning versus conservative treatment for doyle type 4B mallet fractures

**DOI:** 10.1007/s00402-026-06342-z

**Published:** 2026-06-23

**Authors:** Kemal Arda Col, Onur Demirsu, Mahmud Aydın, Serkan Surucu, Murat Yilmaz, Dogan Atlihan

**Affiliations:** 1 Department of Orthopedics and Traumatology, Gaziosmanpaşa Eğitim Ve Araştırma Hastanesi, Istanbul, Turkey; 2 Department of Orthopedics and Traumatology, Haseki Eğitim ve Araştırma Hastanesi, Istanbul, Turkey; 3 Department of Orthopedics and Traumatology, Üsküdar University, Istanbul, Turkey; 4 Department of Orthopedics and Traumatology, Acıbadem Maslak Hospital, Istanbul, Turkey

**Keywords:** Conservative treatment, Crawford criteria, Extension-block pinning technique, Mallet fracture, Pin-orthosis

## Abstract

**Introduction:**

Although numerous treatment options have been reported for mallet fractures, a universally accepted gold-standard approach has not yet been established. The purpose of this study was to compare the clinical and radiographic outcomes of the pin-orthosis extension-block pinning technique (PO-EBPT) with those of conventional conservative treatment in patients with Doyle type 4B mallet fractures.

**Materials and methods:**

This study included 62 patients with Doyle type 4B mallet fractures involving 20–50% of the distal interphalangeal (DIP) joint articular surface, treated between March 2022 and April 2024. Patients were randomized into two groups: Group 1 (*n* = 33) underwent PO-EBPT, whereas Group 2 (*n* = 29) received conservative treatment with splint immobilization. Outcome measures included DIP joint extension lag, range of motion, fracture union, complication rates and functional outcomes according to the Crawford criteria. Follow-up evaluations were performed at 2, 4 and 6 weeks and at 3, 6 and 12 months.

**Results:**

A total of 62 patients were analyzed (33 PO-EBPT; 29 conservative). No statistically significant differences were observed between the groups with respect to sex, affected side, injured finger, or complication rates (*p* = 0.461, *p* = 0.658, *p* = 0.763 and *p* = 0.165, respectively). However, the PO-EBPT group demonstrated significantly improved DIP joint extension lag (4.5 ± 7.8° vs. 12.2 ± 10.4°, *p* = 0.002) and flexion range (88.5 ± 4.4° vs. 86.0 ± 5.7°, *p* = 0.039). According to the Crawford criteria, functional outcomes were also significantly superior in the PO-EBPT group (*p* = 0.02).

**Conclusion:**

PO-EBPT yielded superior functional outcomes compared with conservative treatment in patients with Doyle type 4B mallet fractures, as demonstrated by reduced extension lag, improved DIP joint flexion and higher rates of excellent Crawford scores.

## Introduction

Mallet finger is a deformity of the distal interphalangeal (DIP) joint characterized by an inability to actively extend the fingertip, resulting in persistent flexion of the distal phalanx [1]. This injury typically occurs due to sudden hyperflexion or axial loading of the DIP joint in an extended position, leading either to rupture of the terminal extensor tendon or to an avulsion fracture of the dorsal base of the distal phalanx. When a bony fragment is involved, the injury is defined as a mallet fracture [2–5].

Despite the wide range of treatment options described in the literature, the optimal management of mallet fractures remains controversial [6]. Conservative treatment with splint immobilization is generally recommended for closed injuries without DIP joint subluxation, particularly when the fracture fragment involves less than one-third of the articular surface. However, several studies have reported comparable outcomes between surgical and conservative management even in fractures involving a larger portion of the articular surface [7].

Various surgical techniques have been developed to achieve stable reduction and maintain joint congruity in mallet fractures [7]. Ishiguro’s extension block technique [8], fixation with mini hook plate [9], fixation with pull-out suture [10], fixation with pin-orthosis extension block pinning technique [6, 11], extension block technique with double dorsal K-wire [12], fixation with custom-made plate [3], open reduction K-wire fixation [4] are only a few of the described surgical techniques. Among these, Ishiguro’s extension block pinning technique is widely used because it provides minimally invasive fixation with satisfactory outcomes. Nevertheless, the requirement for transfixation of the DIP joint may lead to complications such as infection, nail deformity and joint stiffness [8]. To overcome these limitations, the pin-orthosis extension-block pinning technique (PO-EBPT) was introduced as a modification that avoids DIP joint transfixation while maintaining fracture reduction using a dorsal Kirschner wire and a volar orthosis [6].

The purpose of this study was to compare the clinical and radiographic outcomes of PO-EBPT with those of conservative treatment in patients with Doyle type 4B mallet fractures. The hypothesis was that PO-EBPT would provide superior functional outcomes due to improved anatomical reduction and stabilization of the fracture fragment.

## Materials and methods

This prospective randomized study was conducted in accordance with the ethical standards of the institutional Clinical Research Ethics Committee and ethical approval was obtained (Decision No 39-2022, Date: 09.03.2022). Patients presenting to the Orthopedics and Traumatology outpatient clinic and emergency department between March 2022 and April 2024 with a diagnosis of Doyle type 4B mallet fracture were screened for eligibility. Written informed consent was obtained from all participants prior to inclusion.

**Fig. 1:**
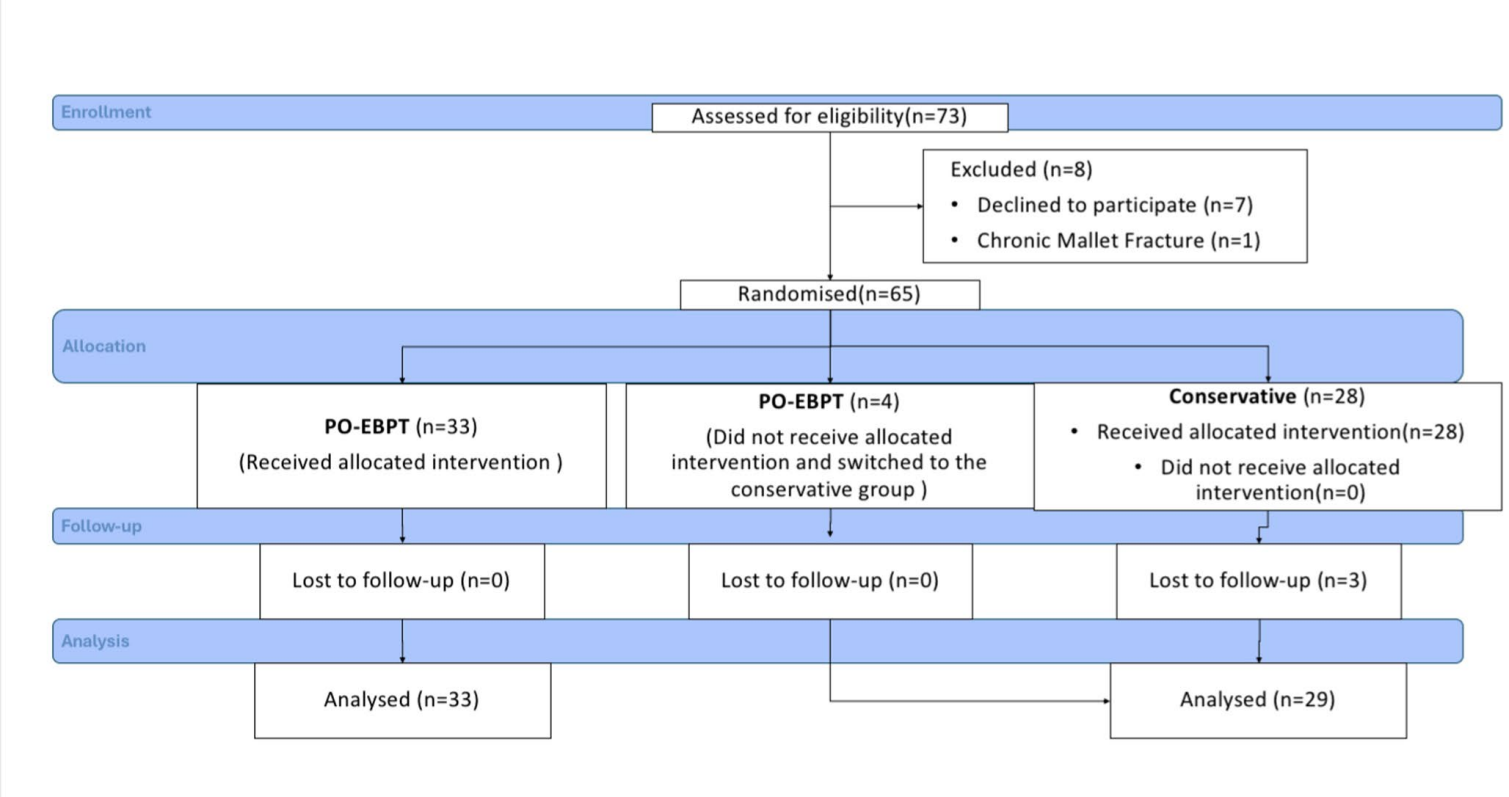
Consort flow diagram

The inclusion criteria were age between 18 and 65 years, acute mallet fracture (< 3 weeks from injury) and fracture fragments involving 20–50% of the distal interphalangeal (DIP) joint articular surface. Exclusion criteria included open fractures, chronic mallet fractures, fracture fragments involving more than 50% of the joint surface, open physes, age younger than 18 years or older than 65 years and failure to complete the 12-month follow-up (Fig. 1).

A total of 62 patients met the inclusion criteria and were enrolled in the study. Patients were randomized using an online randomization tool (random.org) into two treatment groups: the PO-EBPT group (*n* = 33) and the conservative treatment group (*n* = 29). Four patients who declined surgery despite recommendation were allocated to the conservative treatment group and subsequent patients were included in the surgical group to maintain the planned sample size. Three patients in the conservative group were excluded due to loss to follow-up during the 12-month observation period. DIP joint subluxation was defined as volar displacement of the distal phalanx exceeding approximately 7% of the articular surface on lateral radiographs, as previously described [13].

### Conservative technique

**Fig. 2:**
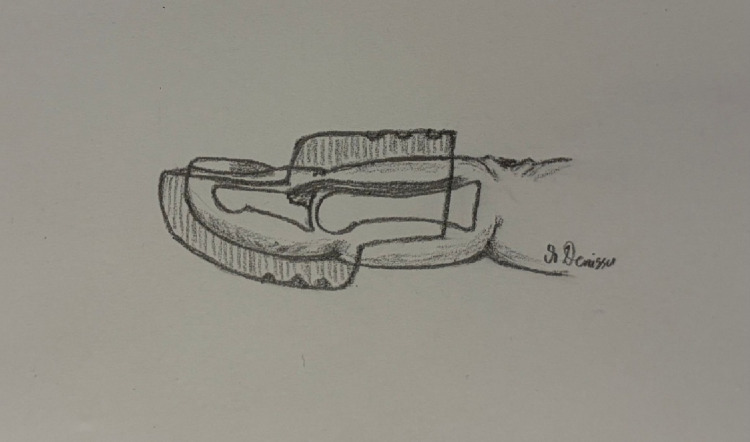
Illustration showing the application of a Stack orthosis with the distal interphalangeal joint maintained in slight hyperextension

Patients assigned to conservative treatment were managed with a Stack orthosis designed to maintain the DIP joint in slight hyperextension while allowing free motion of the proximal interphalangeal joint (Fig. 2). The orthosis was secured with plaster fixation [7, 14]. After application of the orthosis in the clinic and emergency department, fracture reduction was assessed using anteroposterior and lateral radiographs. Satisfactory or acceptable alignment was achieved in all included patients and those with acceptable reduction were managed conservatively. Patients were instructed regarding the importance of strict compliance with continuous splint use and were informed about potential complications related to non-compliance.

The orthosis was worn continuously for six weeks. After this period, part-time splinting was recommended for an additional two weeks during nighttime (approximately 12 h). Follow-up visits were scheduled every two weeks until radiographic evidence of callus formation was observed. During the part-time splinting phase, patients were instructed to begin active DIP joint flexion exercises.

## PO-EBPT surgical technique

All surgical procedures were performed by two orthopedic surgeons under digital nerve block anesthesia with fluoroscopic guidance and without the use of a tourniquet. Prophylactic antibiotic treatment consisting of 1 g cefazolin was administered one hour before surgery. With the DIP joint held in maximal flexion, a 1.2-mm Kirschner wire was inserted into the dorsal aspect of the middle phalanx at approximately 45° relative to the longitudinal axis, targeting the dorsal articular surface proximal to the fracture fragment. Traction was then applied to the distal phalanx and the DIP joint was extended to achieve reduction of the fracture fragment under fluoroscopic guidance.

**Fig. 3:**
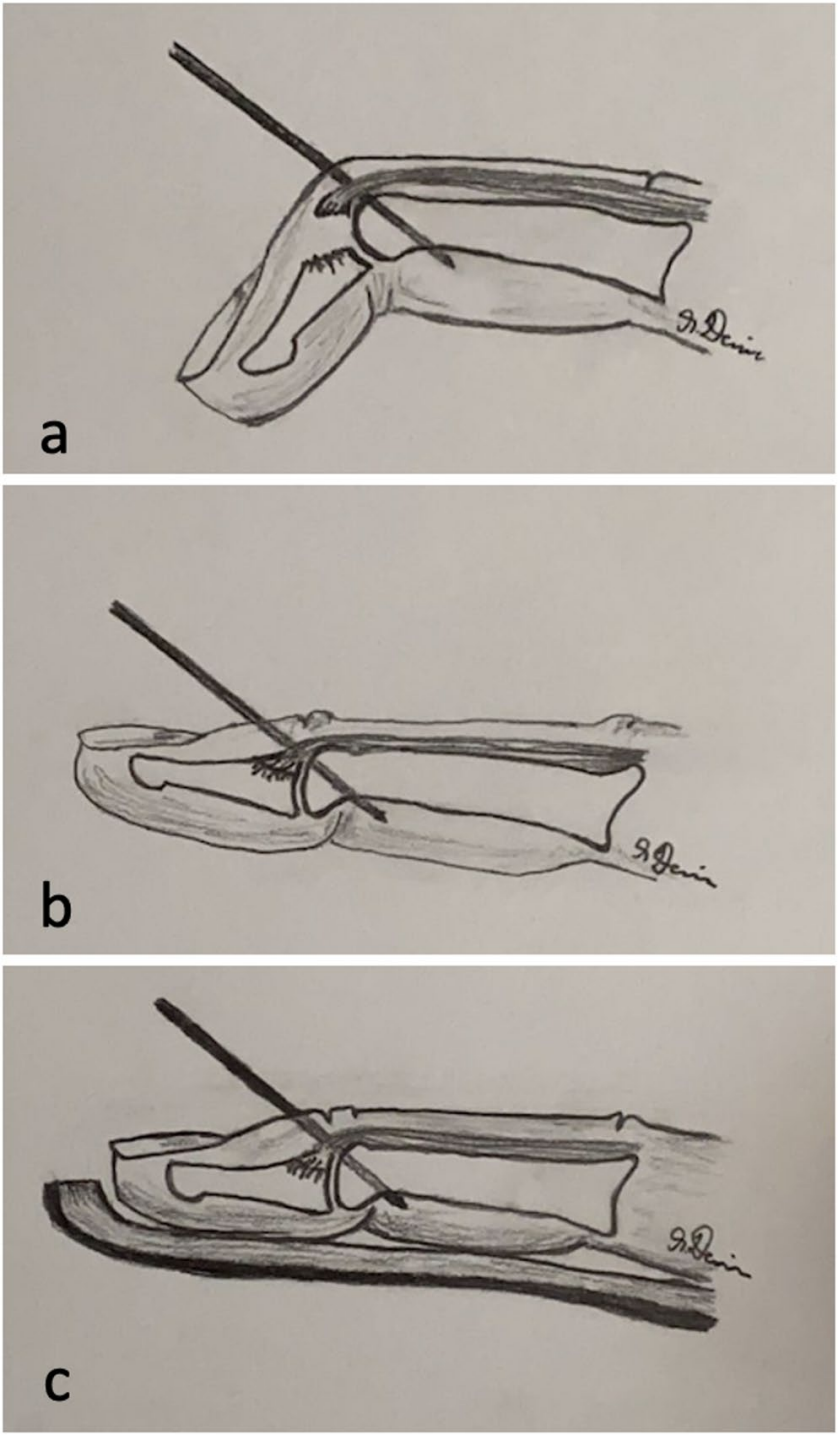
Illustration showing the pin orthosis-extension block pinning technique. a Inserting the first pin into the middle phalanx for indirect reduction while keeping the DIPJ flexed. b DIPJ extension for fracture reduction. c Insertion of the orthosis to hold the DIPJ in the extended position [11]

**Fig. 4:**
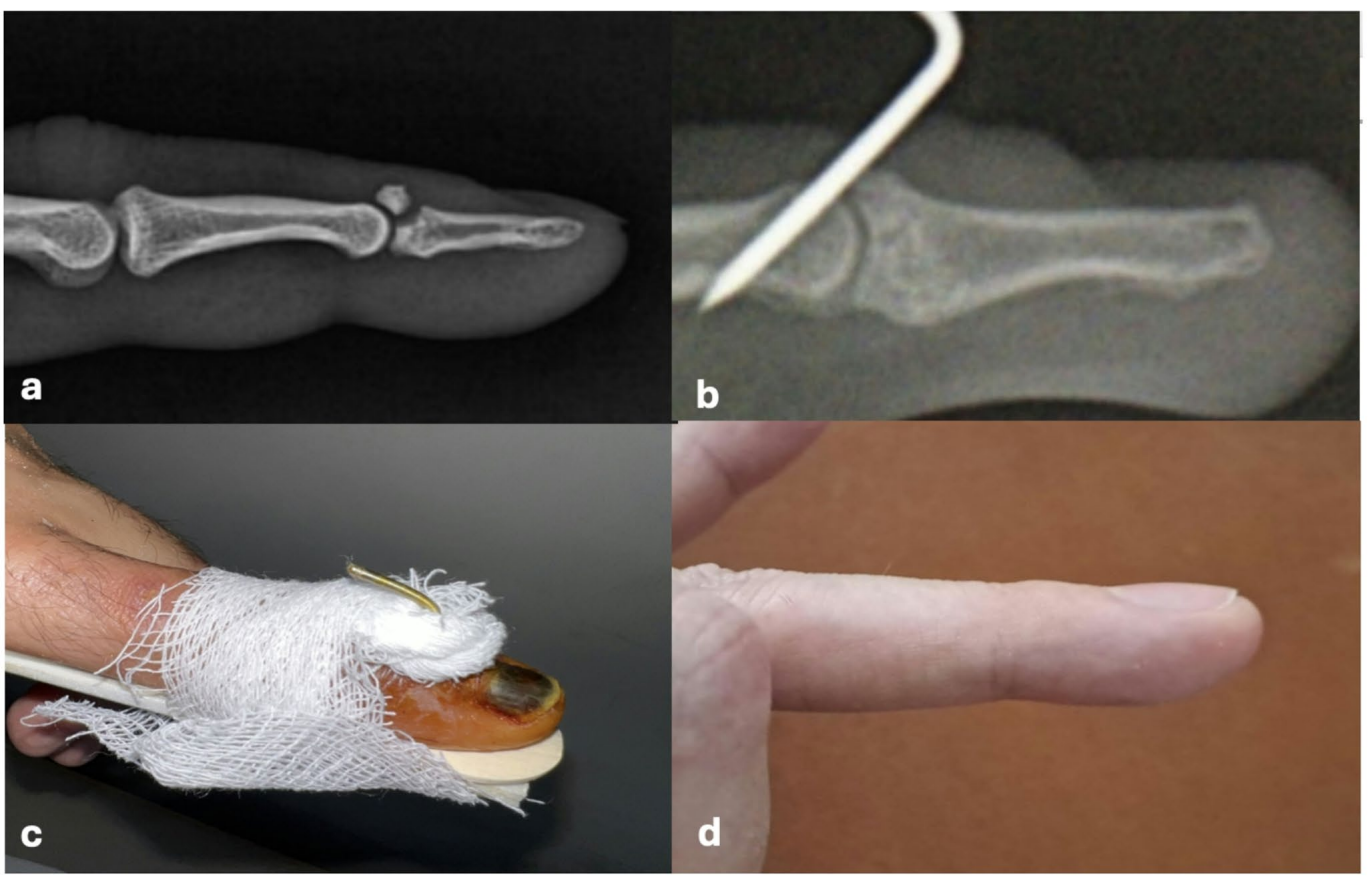
The pin orthosis-extension block pinning technique. **a** Doyle-type 4b mallet fracture lateral radiography image. **b** Postoperative lateral radiography image. **c** Sagittal plane clinical image after orthosis placement. **d** Clinical image at final follow-up demonstrating functional outcome

After confirming adequate reduction, the Kirschner wire was cut approximately 1 cm above the skin level. Sterile strips were applied for skin dressing. (Sterile-Strip; The 3 M Co., Maplewood, MN, USA). A volar aluminum orthosis was then placed to maintain the DIP and proximal interphalangeal joints in extension while allowing motion at the metacarpophalangeal joint (Figs. 3 and 4).

## Postoperative management

Patients were discharged on the day of surgery and followed weekly by the operating surgeon. At each visit, anteroposterior and lateral radiographs were obtained to assess fracture alignment and healing. Dressings were performed with the DIP joint maintained in extension to prevent loss of reduction. The Kirschner wire was removed at the fourth postoperative week under outpatient conditions. Patients continued to use the aluminum orthosis for one additional week. If radiographic evidence of callus formation was insufficient, orthosis use was extended up to six weeks.

Active DIP joint flexion-extension exercises were initiated after orthosis removal in surgically treated patients. In the conservative group, active exercises were initiated after completion of the full-time splinting period.

## Outcome evaluation

At the final follow-up visit, DIP joint extension deficit and active flexion were measured using a finger goniometer and compared with the contralateral finger. Functional outcomes were assessed using the Crawford criteria, which evaluate extension loss, flexion loss and pain [15] (Table 1).

Radiographic evaluations were performed by an independent surgeon during outpatient visits. Standard anteroposterior and lateral radiographs were obtained at the initial visit, immediately after treatment and during follow-up until fracture union was confirmed. Patients were also monitored for complications including dorsal bump formation, non-union, osteoarthritis and persistent extension lag.

**Table 1:** Crawford criteria used for the assessment of functional outcomes

Classification	Extension loss	Flexion	Pain
Excellent	0°	Full	None
Good	1°- 10°	Full	None
Fair	11°- 25°	Any Loss	None
Poor	> 25°	Any Loss	Persistent

### Statistical analysis

Sample size calculation was performed using extension lag as the primary outcome parameter. Assuming a difference of 5° in extension lag between groups with a standard deviation of 5°, a sample size of 26 patients per group provided 95% statistical power with an alpha level of 0.05.

Statistical analyses were performed using SPSS version 27.0. Descriptive statistics were reported as mean, standard deviation, median, minimum, maximum, frequency and percentage. Normality of data distribution was assessed using the Kolmogorov–Smirnov and Shapiro–Wilk tests. The Mann–Whitney U test was used for comparison of non-normally distributed quantitative variables and the chi-square test (Fisher’s exact test when appropriate) was used for categorical variables. A two-sided p value < 0.05 was considered statistically significant.

## Results

A total of 62 patients were included in the study, comprising 20 women and 42 men, with a mean age of 34.0 ± 11.7 years. Patients were divided into two groups according to treatment modality: 29 patients received conservative treatment and 33 patients underwent PO-EBPT. The most common injury mechanism was a fall, accounting for 27 cases (43%) (Table 2).

There were no statistically significant differences between the two groups with respect to sex distribution, injured side, affected finger, complication rate, or injury mechanism. However, the mean age of patients in the PO-EBPT group was significantly lower than that of the conservative group (29.8 ± 9.0 vs. 38.8 ± 12.7 years, *p* = 0.004).

**Fig. 5:**
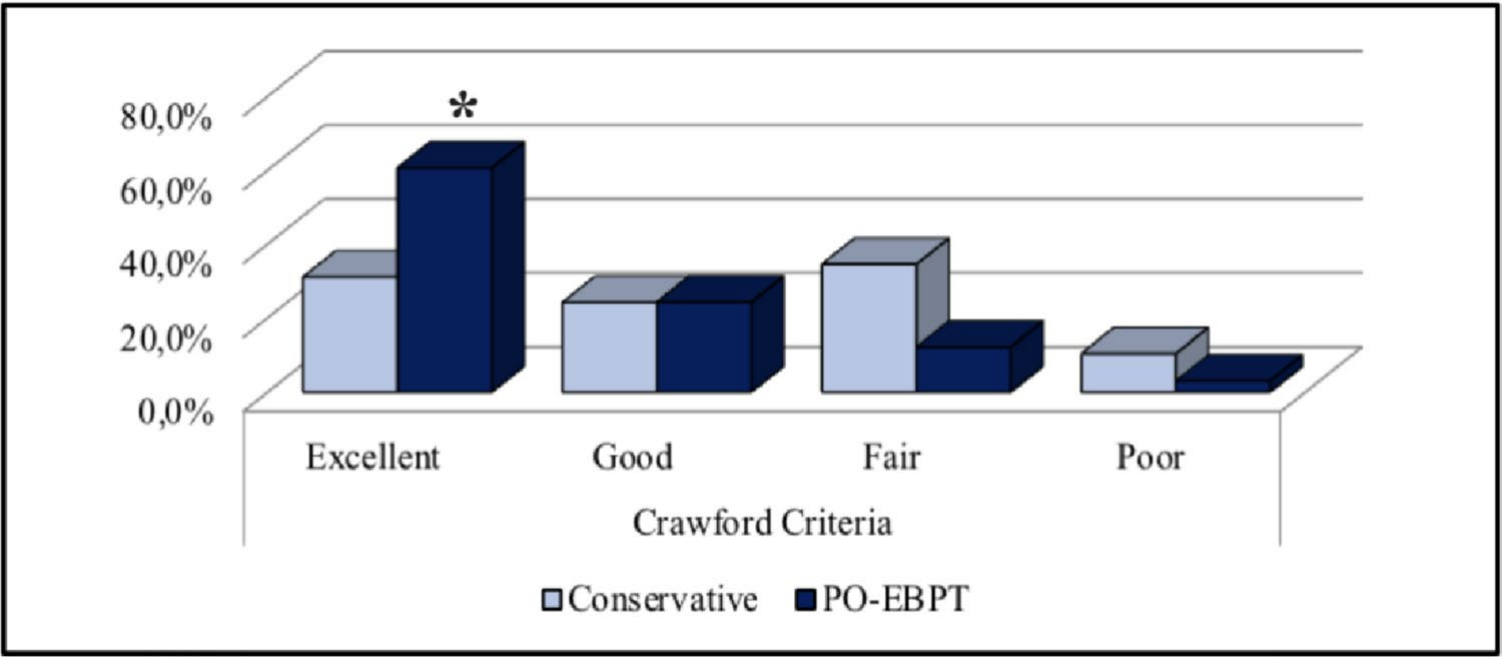
Distribution of patients in both groups according to Crawford Criteria

According to the Crawford criteria, functional outcomes were significantly better in the PO-EBPT group. The number of patients achieving an excellent result was significantly higher in the surgical group compared with the conservative group (20 vs. 9, *p* = 0.02) (Fig. 5).

DIP joint subluxation was significantly more common in the PO-EBPT group than in the conservative group (*p* = 0.032). Subluxation occurred in 14 of 33 patients (42.4%) in the surgical group and in 5 of 29 patients (17.2%) in the conservative group.

Complications occurred in both groups but did not differ significantly in overall frequency. In the conservative treatment group, one patient developed nail deformity, two patients developed osteoarthritis, one patient had both osteoarthritis and nonunion, and one patient reported persistent pain. In the PO-EBPT group, one patient developed skin necrosis and one patient experienced a pin-tract infection.

Radiographic union was observed within three months in all patients except one in the conservative treatment group who developed nonunion. Despite the radiographic finding of nonunion, this patient reported no functional limitation or cosmetic complaint at the final follow-up.

**Table 2:**
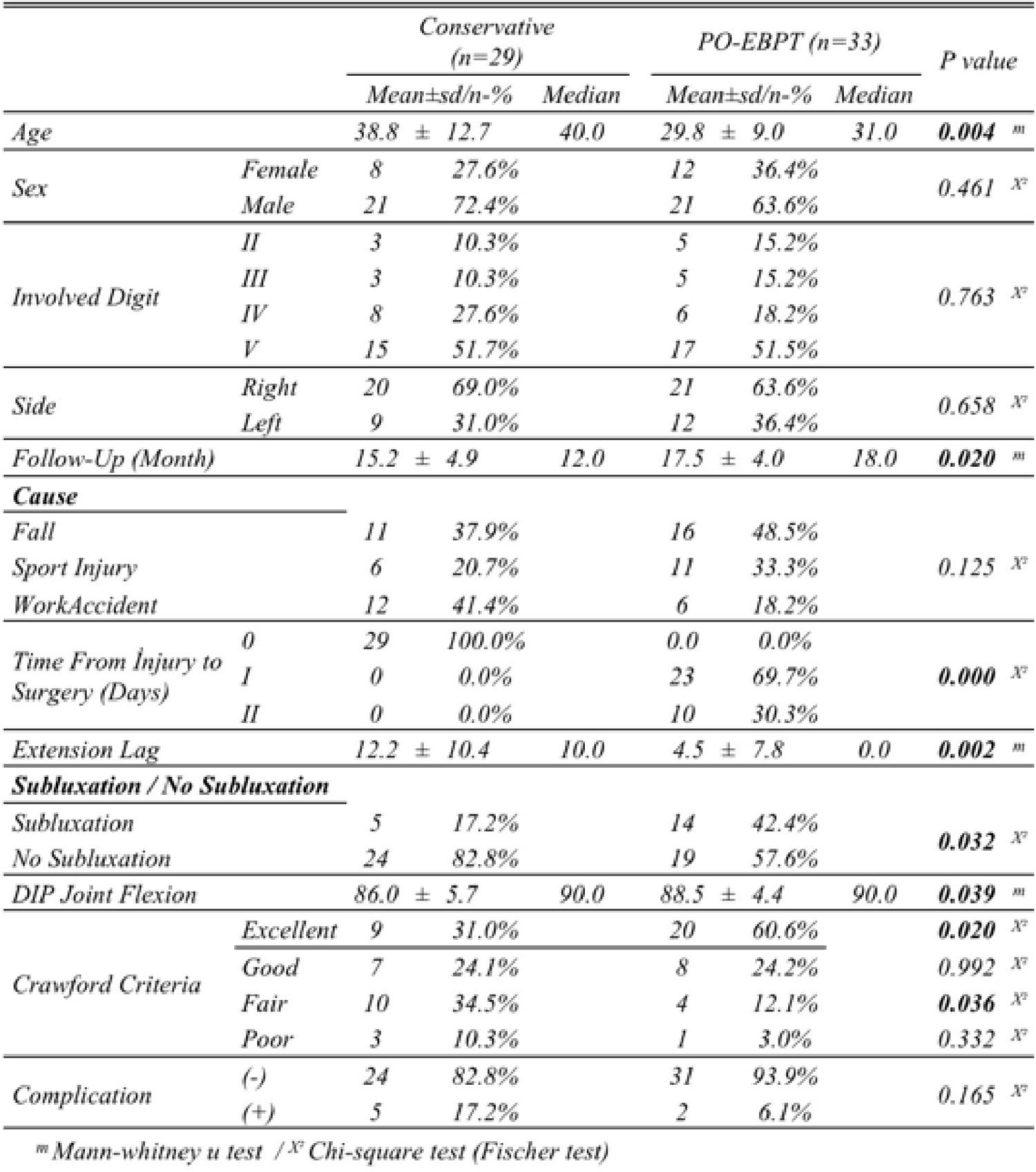
Comparison of postoperative results in both groups

## Discussion

The present study compared the clinical and radiographic outcomes of the pin-orthosis extension-block pinning technique (PO-EBPT) with conservative splint treatment in patients with Doyle type 4B mallet fractures. The main finding of this study was that patients treated with PO-EBPT achieved significantly better functional outcomes than those managed conservatively. Specifically, the surgical group demonstrated significantly lower extension lag, greater DIP joint flexion and a higher proportion of excellent results according to the Crawford criteria.

The optimal treatment of mallet fractures remains controversial. Both conservative splinting and surgical fixation have been widely used, yet a clear consensus regarding the superior treatment modality has not been established [7, 16, 17]. Several authors have suggested that fractures involving more than one-third of the articular surface or those associated with DIP joint subluxation may benefit from surgical intervention [18–20]. However, systematic reviews and meta-analyses have reported comparable outcomes between surgical and conservative management in many cases [21], highlighting the ongoing uncertainty in treatment selection. This outcome has been attributed to four different factors: first, the heterogeneous distribution of patient groups (as various fracture subtypes were examined); second, the insufficient number of patients included in the studies; third, the limited number of investigations addressing this issue; and finally, the restricted scope of the results discussed within these studies.

Conservative treatment remains a widely accepted approach for appropriate mallet fractures due to its simplicity, low cost and generally satisfactory outcomes [7, 17, 22, 23]. Nevertheless, successful conservative management largely depends on strict patient compliance with continuous splint immobilization. Maintaining the DIP joint in extension for several weeks can be challenging, particularly in patients with high functional demands [24, 25]. In addition, prolonged splint use may result in skin-related complications, which may reduce compliance and negatively affect outcomes [26]. In the present study, complications occurred in 17.2% of conservatively treated patients, slightly higher than rates reported in previous meta-analyses (12.8%) [14]. This difference may be explained by the inclusion of fractures involving 20–50% of the articular surface, which represent more advanced injury patterns.

Surgical techniques for mallet fractures aim to achieve stable reduction and restore joint congruity while minimizing soft tissue injury. Among these, Ishiguro’s extension block pinning technique is widely accepted because of its minimally invasive nature and favorable outcomes [8, 27, 28]. However, the requirement for DIP joint transfixation may lead to complications such as infection, nail deformity, joint stiffness and increased radiation exposure during repeated attempts at wire placement. Previous studies have reported an increased risk of early postoperative infection with K-wire fixation although rates may vary depending on the technique [29]. The PO-EBPT technique was developed to overcome these limitations by eliminating the need for DIP joint transfixation while maintaining stable fracture reduction [6]. Previous studies comparing PO-EBPT with the conventional extension block technique have demonstrated superior functional outcomes and reduced extension loss in patients treated with PO-EBPT [11]. The findings of the present study are consistent with these reports and further support the clinical effectiveness of this technique.

The superior outcomes observed in the PO-EBPT group may be explained by several factors. First, surgical fixation enables anatomical reduction of the intra-articular fracture fragment, which may help preserve joint congruity and improve functional outcomes. Second, stabilization with the Kirschner wire during the early postoperative period, followed by continued use of an aluminum orthosis to maintain DIP joint extension, may help maintain reduction of the fracture fragment throughout the healing process [14, 30]. In contrast, patients treated non-operatively may experience fragment rotation or loss of reduction, particularly in larger fractures. Although some authors have suggested that anatomical reduction may not be essential in mallet fractures due to the non-weight-bearing nature of the DIP joint and its remodeling potential [29], the present findings indicate that improved reduction and stabilization may translate into better functional outcomes. Notably, none of the patients treated with PO-EBPT developed osteoarthritis or persistent pain during the follow-up period. In addition, DIP joint subluxation was more frequently observed in the PO-EBPT group. This finding is clinically relevant, as previous clinical and biomechanical studies have suggested that subluxation of the distal phalanx may be associated with an increased risk of degenerative joint changes, including osteoarthritis [13, 31] The higher rate of DIP joint subluxation observed in the PO-EBPT group warrants further consideration. One possible explanation may be related to the postoperative immobilization technique. Unlike prefabricated Stack orthoses, the degree of extension in the aluminum orthosis is manually adjusted and excessive extension or overcorrection may lead to volar displacement of the distal phalanx. Despite the higher incidence of subluxation, functional outcomes in the surgical group were superior. This suggests that mild degrees of subluxation may not necessarily correlate with worse clinical outcomes in the short term, although their potential role in the development of long-term degenerative changes should be considered.

Recent studies investigating alternative surgical techniques have also reported favorable outcomes. Guo et al. demonstrated excellent or good results using combined K-wire and pull-out wire fixation, although the study was limited by a small sample size and short follow-up [32]. Similarly, Yue et al. reported predominantly excellent outcomes using dorsal K-wire reduction combined with volar splint fixation, although the lack of a control group limits interpretation [33]. Abubeih et al. reported good functional outcomes with modified hook-plate fixation but noted a relatively high complication rate of 35% [34]. These findings highlight the ongoing efforts to optimize surgical management while minimizing complications. Despite the presence of radiographic non-union in one patient in our study, no functional limitation was observed. This finding is consistent with evidence suggesting that radiographic non-union in distal phalanx fractures may not adversely affect functional outcomes [35].

This study has several limitations. First, age > 65 years was used as an exclusion criterion to minimize the potential confounding effect of age-related degenerative changes. However, the absence of a standardized radiographic assessment of osteoarthrosis represents a limitation. Second, although randomization was performed, the mean age of the conservative group was higher than that of the surgical group. However, previous studies have suggested that age does not significantly affect outcomes in mallet finger injuries [10]. Third, the sample size was relatively small and the study was conducted at a single center, which may limit generalizability. Fourth, functional evaluation was based solely on the Crawford criteria and did not include patient-reported outcome measures such as DASH or QuickDASH scores. Finally, although follow-up extended to 12 months, longer-term studies are needed to assess late degenerative changes. Despite these limitations, the study has several strengths. The prospective randomized design reduces selection bias and enhances internal validity. The inclusion of a homogeneous patient population limited to Doyle type 4B fractures allowed for a more focused comparison. In addition, the use of a priori power analysis ensured adequate statistical strength. To our knowledge, this study represents one of the few prospective randomized trials directly comparing PO-EBPT with conservative treatment in this specific fracture subtype.

## Conclusion

PO-EBPT demonstrated superior functional outcomes compared with conservative treatment in patients with Doyle type 4B mallet fractures, as evidenced by reduced extension lag, improved DIP joint flexion, and higher rates of excellent results according to the Crawford criteria.

## Data Availability

The datasets generated and/or analyzed during the current study are not publicly available due to patient privacy concerns but are available from the corresponding author upon reasonable request.

## Abbreviations

PO-EBPT: Pin Orthosis extension-block pinning technique
DIP: Distal interphalangeal
